# Role of capsule endoscopy in suspected celiac disease: A European multi-centre study

**DOI:** 10.3748/wjg.v23.i4.703

**Published:** 2017-01-28

**Authors:** Marisol Luján-Sanchis, Enrique Pérez-Cuadrado-Robles, Javier García-Lledó, José-Francisco Juanmartiñena Fernández, Luca Elli, Victoria-Alejandra Jiménez-García, Juan Egea-Valenzuela, Julio Valle-Muñoz, Cristina Carretero-Ribón, Ignacio Fernández-Urién-Sainz, Antonio López-Higueras, Noelia Alonso-Lázaro, Mileidis Sanjuan-Acosta, Francisco Sánchez-Ceballos, Bruno Rosa, Santiago González-Vázquez, Federica Branchi, Lucía Ruano-Díaz, César Prieto-de-Frías, Vicente Pons-Beltrán, Pilar Borque-Barrera, Begoña González-Suárez, Sofía Xavier, Federico Argüelles-Arias, Juan-Manuel Herrerías-Gutiérrez, Enrique Pérez-Cuadrado-Martínez, Javier Sempere-García-Argüelles

**Affiliations:** Marisol Luján-Sanchis, Javier Sempere-García-Argüelles, Digestive Diseases Unit, General University Hospital of Valencia, 46026 Valencia, Spain; Enrique Pérez-Cuadrado-Robles, Antonio López-Higueras, Enrique Pérez-Cuadrado-Martínez, Small Bowel Unit, Hospital Morales Meseguer, 30008 Murcia, Spain; Javier García-Lledó, Digestive Diseases Unit, General University Gregorio Marañón, 28007 Madrid, Spain; José-Francisco Juanmartiñena Fernández, Ignacio Fernández-Urién-Sainz, Unit of Gastroenterology and Endoscopy, Complejo Hospitalario de Navarra, 31008 Pamplona, Spain; Luca Elli, Federica Branchi, Center for prevention and Diagnosis of Celiac Disease, Fondazione IRCCS Ca’ Granda Ospedale Maggiore Policlinico, 20122 Milan, Italy; Victoria-Alejandra Jiménez-García, Federico Argüelles-Arias, Juan-Manuel Herrerías-Gutiérrez, Unit of Gastroenterology and Endoscopy, University Hospital Virgen Macarena, 41071 Sevilla, Spain; Juan Egea-Valenzuela, Gastroenterology department, Hospital Virgen de la Arrixaca, 30120 Murcia, Spain; Julio Valle-Muñoz, Lucía Ruano-Díaz, Department of Gastroenterology, Complejo Hospitalario de Toledo, 45005 Toledo, Spain; Cristina Carretero-Ribón, Santiago González-Vázquez, César Prieto-de-Frías, Department of Gastroenterology, University of Navarra Clinic, 31009 Pamplona, Spain; Noelia Alonso-Lázaro, Vicente Pons-Beltrán, Endoscopy Digestive Unit, Digestive Diseases Area, Universitari i Politècnic La Fe Hospital, 46026 Valencia, Spain; Mileidis Sanjuan-Acosta, Pilar Borque-Barrera, Digestive Diseases Unit, University Hospital Nuestra Señora de Candelaria, 38010 Tenerife, Spain; Francisco Sánchez-Ceballos, Digestive Diseases Unit, Clinical Hospital San Carlos, 28040 Madrid, Spain; Bruno Rosa, Sofía Xavier, Digestive Diseases Unit, Hospital da Senhora da Oliveira - Guimarães, 114 Cutileiros, Portugal; Begoña González-Suárez, Endoscopy Digestive Unit, Hospital Clinic de Barcelona, 08036 Barcelona, Spain

**Keywords:** Capsule endoscopy, Celiac disease, Anti-transglutaminase antibodies, Gluten-free diet, Non-celiac gluten sensitivity

## Abstract

**AIM:**

To analyze the diagnostic yield (DY), therapeutic impact (TI) and safety of capsule endoscopy (CE).

**METHODS:**

This is a multi-centre, observational, analytical, retrospective study. A total of 163 patients with suspicion of celiac disease (CD) (mean age = 46.4 ± 17.3 years, 68.1% women) who underwent CE from 2003 to 2015 were included. Patients were divided into four groups: seronegative CD with atrophy (Group-I, *n* = 19), seropositive CD without atrophy (Group-II, *n* = 39), contraindication to gastroscopy (Group-III, *n* = 6), seronegative CD without atrophy, but with a compatible context (Group-IV, *n* = 99). DY, TI and the safety of CE were analysed.

**RESULTS:**

The overall DY was 54% and the final diagnosis was villous atrophy (*n* = 65, 39.9%), complicated CD (*n* = 12, 7.4%) and other enteropathies (*n* = 11, 6.8%; 8 Crohn’s). DY for groups I to IV was 73.7%, 69.2%, 50% and 44.4%, respectively. Atrophy was located in duodenum in 24 cases (36.9%), diffuse in 19 (29.2%), jejunal in 11 (16.9%), and patchy in 10 cases (15.4%). Factors associated with a greater DY were positive serology (68.3% *vs* 49.2%, *P* = 0.034) and older age (*P* = 0.008). On the other hand, neither sex nor clinical presentation, family background, positive histology or HLA status were associated with DY. CE results changed the therapeutic approach in 71.8% of the cases. Atrophy was associated with a greater TI (92.3% *vs* 45.3%, *P* < 0.001) and 81.9% of the patients responded to diet. There was one case of capsule retention (0.6%). Agreement between CE findings and subsequent histology was 100% for diagnosing normal/other conditions, 70% for suspected CD and 50% for complicated CD.

**CONCLUSION:**

CE has a high DY in cases of suspicion of CD and it leads to changes in the clinical course of the disease. CE is safe procedure with a high degree of concordance with histology and it helps in the differential diagnosis of CD.

**Core tip:** We present the experience of 14 European centers in the indication of impact of capsule endoscopy for suspected celiac disease. It is the study with more patients published to date. We describe the diagnostic and therapeutic impact of capsule in celiac disease, as well as the safety of the technique for this indication.

## INTRODUCTION

Celiac disease (CD) is the most common autoimmune enteropathy[1], and it is characterized by gluten-induced chronic inflammation of the small bowel (SB). The diagnosis of CD requires the analysis of clinical, histopathological, and serological factors. Genetic factors are not performed routinely, they only help in dubious cases. These has a role primarily exclusion of this diagnosis by its high negative predictive value. Currently, serology anti-transglutaminase antibodies (ATG) is the test of choice for the initial diagnosis and monitoring[2,3], although gaved by false positives and negatives[4]. The presence of villous atrophy on duodenal biopsy (DB) through upper digestive endoscopy remains the gold standard for diagnosis in adults, although patchy intestinal impairment can yield to false negatives[5]. Thus, there are a significant proportion of patients without classic CD diagnostic criteria who present with discordant data and pose a diagnostic challenge. Today allowed 4 of the 5 criteria Salerno[6]: clinical, high titer serology, HLA feature biopsy and/or clinical and histological response to gluten-free diet (GFD). Seronegative cases have led to the recent description of a spectrum of diseases related to gluten, such as seronegative CD and non-celiac gluten sensitivity (NCGS)[7]. The prevalence of NCGS is high, as shown in a multi-centre randomized and controlled study, which found NCGS in 14% of 140 patients with functional gastrointestinal symptoms. The absence of SB damage is necessary to suspect a NCGS, thus, in presence of atrophy it is impossible to diagnose of NCGS[8]. These new nosological concepts help to classify patients who do not meet the classic criteria and could benefit from a GFD[9].

**Figure 1:**
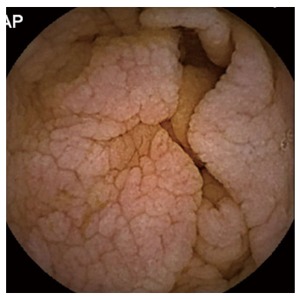
Image suggestive of celiac disease by capsule endoscopy.

Capsule endoscopy (CE) is an endoscopy technique, which visualizes the entire SB and has proven useful in patients with negative serology and intestinal atrophy or Marsh-I/II[10]. The most common signs compatible with CD are the reduction or absence of Kerckring folds (65%), followed by scalloping (55%) and a mosaic pattern with nodularity (32%)[10] (Figure 1). This technique can detect villous atrophy with greater sensitivity than conventional endoscopy (92% *vs* 55%)[11] and it has demonstrated high cost effectiveness and diagnostic accuracy for CD[12-14]. However, recent European guidelines[15] relegate the role of CE to an alternative for patients who do not want or cannot undergo conventional endoscopy[16], possibly due the shortage of relevant publications, which also comprise low numbers of cases. Therefore, our objective was to analyse the impact of CE in a European multi-centre study of patients with suspected CD who do not meet the classic CD criteria or who have discordant results in common diagnostic tests.

## MATERIALS AND METHODS

### Patients and definitions

An analytical retrospective observational study involving 14 hospitals in Europe. This article was coordinated by the Valencia University General Hospital Consortium. It has been approved by the local institutional review board and approved by its Ethics Committee.

**Figure 2:**
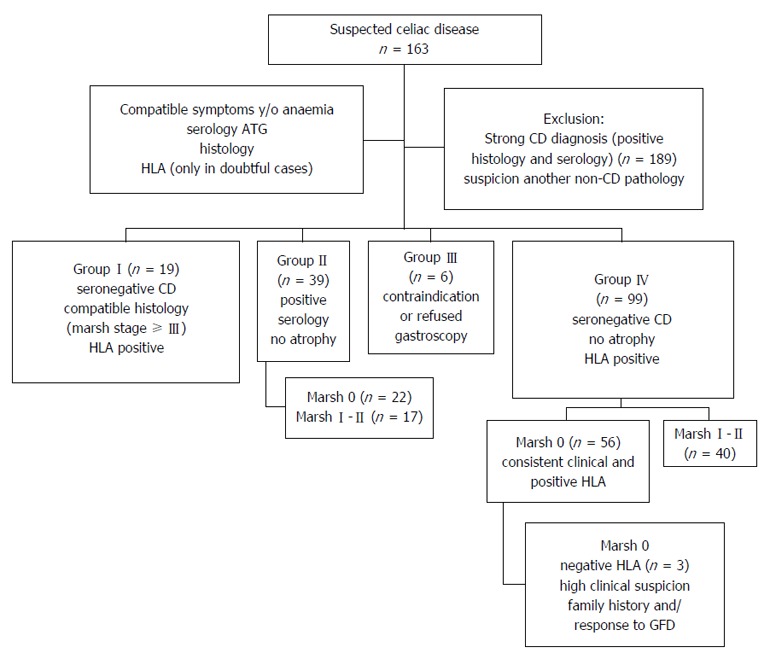
Groups with suspected celiac disease included and excluded in the study. CE: Capsule endoscopy; CD: Celiac disease; ATG: Anti-transglutaminase antibodies; GFD: Gluten-free diet.

**Inclusion criteria:** An analysis of the clinical histories of 163 patients (mean age: 46.4 ± 17.3 years, range: 11-85; 68.1% women) was conducted; the patients underwent CE for suspected CD during the years 2003-2015. The suspicion of CD was based on a clinical assessment indicating symptoms that were compatible with CD, serology (ATG and to rule IgA deficit) and histology. In selected cases HLA status DQ2/DQ8 (exclusively in doubtful cases). Family history of CD were collected. All patients underwent gastroscopy to determine the basal histology except when endoscopy was contraindicated. Depending on the protocol for each centre, there were 2-6 DB (of the bulb and/or second duodenal portion). Intestinal damage was calculated using the Marsh classification[17], with stages III and higher considered positive. Based on the various findings, patients were classified into 4 suspect groups, as follows (Figure 2): (1) Group-I (*n* = 19): Seronegative CD. Patients with negative serology, histology compatible with CD and positive HLA; (2) Group-II (*n* = 39): Patients with positive serology, no atrophy and Marsh stages 0 (*n* = 22) or I-II (*n* = 17); (3) Group-III (*n* = 6): Patients with contraindications or refusal to undergo gastroscopy; and (4) Group-IV (*n* = 99): Seronegative patients without atrophy, with clinical digestive symptoms and/or anaemia. Studied according to their histology, which indicated Marsh stages 0 (*n* = 59) or I-II (*n* = 40). Patients with Marsh 0, showed consistent clinical and positive HLA. Patients with Marsh 0 and negative HLA (*n* = 3) were included by high clinical suspicion, other family members and/or to advise those who need for accurate diagnosis for response to GFD and need to maintain or withdraw it (Figure 2).

**Exclusion criteria:** Patients with a strong CD diagnosis (positive histology and serology) were excluded (*n* = 189), as were those who underwent CE due to suspicion of another non-celiac pathology, or requests for obscure gastrointestinal bleeding without other data suspicion of CD. Those who presented with negative serology and histology without compatible clinical/analytical characteristics (regardless of the HLA), and those who were HLA negative when the only suspect data point was clinical presentation.

### Procedure

Multiple CE systems were used, including *Pillcam SB, SB2, SB3, COLON 1 and COLON 2 (Medtronic Inc, Dublin, Ireland) and Mirocam (Intromedic, Seoul, Korea*). Prior capsule patency was indicated in 6 patients (3.7%) and was normal in all cases. The indication of the type of CE, patency and the prior preparation followed the protocol of each centre. The location of the lesions and their extent in the various SB segments were recorded.

**Figure 3:**
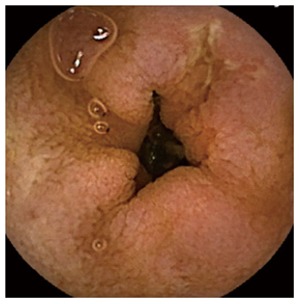
Image suggestive of celiac disease complicated by ulcerative jejunitis.

The diagnostic yield (DY) of CE was considered positive when CE found pathological findings (nodular mucosa, mosaic pattern, villous atrophy, scalloping folds) (Figure 1), either intestinal atrophy, complications from CD (ulcerative lesions mainly in jejunum-Figure 3, neoformation), or diagnoses than CD. The distinction between CD and other enteropathies was made by each center based on clinical, analytical, radiological, endoscopic and response criteria to specific treatments.

The therapeutic impact (TI) was considered positive when the CE changed the therapeutic approach or the patient’s evolutionary course, including the modifications of a GFD or a specific treatment for CD or other enteropathies or subsequent digestive endoscopies. Both impacts were analysed based on the different groups previously described. When the CE indicated the implementation of new endoscopic procedures with biopsy, the agreement with histology was analysed.

### Statistical analysis

Categorical variables were compared using a χ^2^-test or Fisher’s test. Normally distributed continuous variables were presented as the mean, standard deviation and analysed by a Student *t*-test. *P*-values < 0.05 were considered statistically significant. SPSS version 23 was used (IBM, SPSS Inc., IL, United States).

## RESULTS

### Patients

The overall prevalence of positive serology by ATG (*n* = 38) or anti-gliadin/endomysium antibodies (*n* = 3) was 25.15%. The HLA was positive (*n* = 68, 41.7%), negative (*n* = 8, 4.9%) and in most it was not performed (*n* = 86, 52.8%). All cases of negative HLA (three Marsh 0, four Marsh-I and one Marsh-II) corresponded to the patients in Group-IV.

The presentation forms were clinical digestive symptoms (*n* = 95, 58.3%), iron-deficiency anaemia or iron deficiency (*n* = 22, 13.5%), or both (*n* = 42, 25.8%). In addition, the associated dermatitis herpetiformis (*n* = 4, 2.5%), neurological syndromes (*n* = 3, 1.8%, one in the form of ataxia with suspected Gobbi syndrome) and stunted growth (*n* = 2, 1.2%) were found. The family history of CD (*n* = 11, 6.8%) was also collected.

### Diagnostic yield of capsule endoscopy

The average SB transit time was 232.1 ± 89.9 min, with full visualization in 92.6% cases. There were 6 incomplete procedures, and 50% of them reached a diagnosis. There was only one complication (0.6%) due to retention of the CE secondary to ulcerative jejunitis (UJ), and the CE was extracted by balloon-assisted enteroscopy (BAE), which confirmed the diagnosis.

Overall, the DY of the CE diagnosis was 54% (*n* = 88). The DY obtained by the subgroups is shown in Table 1. The CE results were suggestive of intestinal atrophy (*n* = 65, 39.9%), UJ (Figure 3) (*n* = 11, 6.8%), intestinal lymphoma in the jejunum (*n* = 1, 0.6%) and other enteropathies (*n* = 11, 6.8%). Positive serology (68.3% *vs* 49.2%, *P =* 0.034) and age (50 ± 17 *vs* 43 ± 17, *P = 0.008*) were associated with a larger impact on diagnosis, but positive histology at baseline (73.7% *vs* 51.5%, *P =* 0.068), HLA (60.3% *vs* 55.6%, *P =* 0.785, sex (*P =* 0.717), clinical presentation (*P =* 0.993) and family background (*P =* 0.745) were not. In seropositive patients (Group-II), there were no differences between those with DY statuses for the Marsh-0 and Marsh-I/II stages (59.1% *vs* 82.4%, *P =* 0.119).

**Table 1:** Diagnostic performance of capsule endoscopy for the subgroups

**Subgroup (*n*, %yield)^1^**	**Normal**	**Intestinal atrophy**	**Complicated CD**	**Other enteropathies**
I (*n* = 14/19, 73.7%)	5 (26.3)	9 (47.4)	5 (26.3)	0
II (*n* = 27/39, 69.2%)	12 (30.8)	25 (64.1)	1 (2.6)	1 (2.6)
III (*n* = 3/6, 50%)	3 (50.0)	3 (50.0)	0	0
IV (*n* = 44/99, 44.4%)	55 (55.6)	28 (28.3)	6 (6.1)	10 (10.1)

1 Group-I: Seronegative celiac disease; Group-II: Positive serology with no atrophy; Group-III: Contraindications or refusal to undergo gastroscopy; Group-IV: Seronegative patients without atrophy, with clinical digestive symptoms and/or anaemia. CD: Celiac disease.

The atrophy was exclusively duodenal (*n* = 24, 36.9%), jejunal (*n* = 11, 16.9%), or ileal (*n* = 1, 1.5%), was diffuse in at least 2 areas (*n* = 19, 29.2%) and was patchy (*n* = 10, 15.4%). The diagnosis of atrophy was associated with a greater TI than when the CE result was normal (92.3% *vs* 45.3%, *P* < 0.001). Three patients with UJ also presented duodenal involvement, and 3 were exclusively ileal. In a case when the UJ affected the entire SB, a sprue-like enteropathy associated with olmesartan was eventually confirmed. Of these patients, at least 2 initially presented with digestive symptoms and had negative serology. Patient who had positive serology and a biopsy indicating Marsh-I, was diagnosed with suspected of jejunal lymphoma by CE; however, subsequent biopsies using BAE did not confirm this finding and showed intestinal atrophy corresponding to Marsh-III. The CE results indicated diagnoses of non-CD enteropathies, mostly in Group-IV (*n* = 11, 6.7%) and Group-II (*n* = 1, 2.6%). The most frequent was Crohn's disease (*n* = 8, 72.7%), and the location was exclusively jejunal (*n* = 3), duodenojejunal (*n* = 1), jejunoileal (*n* = 2) and exclusively ileal (*n* = 1). A stenosis was detected by CE in a patient with jejunal Crohn's, but the capsule could still pass through the stenosis. Only one patient was finally confirmed as having Crohn's disease and associated CD; this patient responded positively to corticosteroids and GFD. In addition, one patient was diagnosed with proctosigmoiditis (ulcerative colitis) through colon CE and a SB intussusception associated with non-specific enteritis and an enteropathy treated with nonsteroidal anti-inflammatory drugs.

### Therapeutic impact of capsule endoscopy

The global TI was 71.8% (*n* = 117), with the suggested changes including that a GFD should be used (*n* = 85, 72.7%) or should be stopped (*n* = 4, 3.4%) and that specific drugs should be used (*n* = 24, 20.5%). In addition, further endoscopy was suggested by the CE results in 36 cases (30.8%), including BAE (*n* = 18), new gastroscopy (*n* = 15) and ileocolonoscopy for Crohn (*n* = 3). One patient was diagnosed with T-cell intestinal lymphoma of the jejunum, and BAE with biopsy was indicated after the discovery of diffuse intestinal atrophy by CE. There were 18 BAE following CE results. However, half of the cases (*n* = 9) were carried out in patients presenting with complicated CD by CE or suspected Crohn disease and only 9 presented with atrophy by CE. Thus, the impact of BAE *vs* conventional endoscopy in this setting can not be concluded because of the low number of cases.

The CE results agreed with the endoscopy results when endoscopy was suggested (Table 2); villous atrophy suggestive of CD agreed in 70% of the cases. The impact for the subgroups and the therapeutic response to the indicated GFD are shown in Table 3. Overall, 81.2% of the patients responded to the GDF. In the two cases that did not show a response, autoimmune enteropathy was diagnosed after evidence of villous atrophy was found by the CE. Only one patient worsened after the withdrawal of the GFD. In the other three cases, the non-responders (Group-IV) showed no atrophy, as observed by CE. The response to specific drugs was 58.3%. Complications from the CD and other enteropathies, such as Crohn’s disease, were treated according to the usual protocol of each centre. Of the patients with normal CE results, 29.3% (*n* = 22/75) responded to the GFD, whereas 63.1% (*n* = 41/65) of the patients for whom atrophy was observed by CE responded to the GFD; this difference was significant (*P <* 0.001). Of the 41 patients in Group-IV, 95.1% responded favourably to the GFD, without a significant difference in the response between Marsh-I/II (*n* = 21) and Marsh-0 (*n* = 20) (90.5% *vs* 100%, *P = 0.488*). NCGS was diagnosed in symptomatic patients of Group-IV (seronegative CD without atrophy) when they clinically responded to the GFD (*n* = 15/39, 38.5%), which was started after normal CE results without any further confirmation of classic CD.

**Table 2:** Comparison of capsule endoscopy results with a subsequent histology evaluation of the same patient

**CE diagnostic yield**	**Biopsied cases**	**Biopsy result**	**Agreement**
Normal	*n* = 2	Normal (*n* = 2)	100%
Atrophy	*n* = 10	Normal (*n* = 2), atrophy (*n* = 7)^2^, lymphoma (*n* = 1)	70%
Complicated CD^1^	*n* = 4	Atrophy (*n* = 2), UJ (*n* = 2)	50%
Other diagnoses	*n* = 3	Crohn’s disease (*n* = 2),	100%
		Ulcerative colitis (*n* = 1)	

1 Three ulcerative jejunoileitis (UJ) and one lymphoma;

2 One of the biopsies reported Marsh III and eosinophilic gastroenteropathy. CE: Capsule endoscopy; CD: Celiac disease.

**Table 3:** Modifications and response to the gluten-free diet in the different subgroups of patients *n* (%)

**Subgroup^1^**	**Therapeutic yield**	**GFD Indicated**	**GFD removed**	**Responded to GFD**	**Responded to the withdrawal**
I (*n* =19)	16 (84.2)	12 (63.2)	1 (5.3)	8 (66.7)	0
II (*n* = 39)	31 (79.5)	27 (69.2)	0	18 (66.7)	-
III (*n* = 6)	6 (100)	5 (83.3)	0	4 (80)	-
IV (*n* = 99)	64 (64.7)	41 (41.4)	3 (3.1)	39 (95.1)	3 (100)

1 Group-I: seronegative celiac disease; Group-II: Positive serology with no atrophy; Group-III: Contraindications or refusal to undergo gastroscopy; Group-IV: Seronegative patients without atrophy, with clinical digestive symptoms and/or anaemia. GFD: Gluten-free diet.

## DISCUSSION

The present multi-centre study describes a series of 163 patients with suspected CD in whom CE was performed in the absence of traditional diagnostic evidence. In these cases, the CE is the first non-invasive alternative diagnostic test in SB when suspicion is high[18]. The global DY of CE was beneficial for more than half of the cases. The most frequent finding was intestinal atrophy, followed by complicated CD and other enteropathies. Most of the patients included presented with clinical symptoms, with positive serologic markers and negative atrophy. In nearly 40% of them, when the CE result was normal, NCGS could be diagnosed. Likewise, more than half of seropositive patients without atrophy were diagnosed as having CD. The CE results influenced the therapeutic approach or evolutionary course in approximately 70% of the cases, and in most of them, the patients responded to the GFD.

The sensitivity and specificity of CE in CD is 83%-89% and 95%-98%, respectively[13,14], and CE is a cost-effective technique for the diagnosis of intestinal atrophy. In addition, CE allows a differential diagnosis to be performed based on observations of the entire SB. In patients with suspected CD, CE is indicated when conventional endoscopy is contraindicated or refused; this technique was initially used in cases of refractory CD and in cases of suspected complications[15,19,20]. Another potential indication includes the ambiguous cases of CD that show disagreement between serology and histology.

In our study, the diagnostic performance was greater in patients with positive serology and in the seronegative patients with positive histology. HLA, sex, clinical presentation and family background were not associated with CD and cannot serve as a guide for indicating CE. In patients with seronegative CD (Group-I), the absence of antibodies could be associated with fluctuating antibodies, advanced age or a GFD[4]. The negative serology is inversely related to the degree of atrophy because this entity includes initial states (latent and potential atypical CD)[21], reducing the benefits of CE[22]. However, in our study, CE is very beneficial in these cases because CE has a high global diagnosis effect, providing a diagnosis of villous atrophy in almost half of the patients and in a significant proportion of the patients who had complicated CD. Other authors have demonstrated that the benefits of CE will be greater for patients corresponding to Marsh-III *vs* Marsh-I-II (28% *vs* 7%)[10]. However, in our study, the presence of villous atrophy at baseline was not significantly associated with a higher DY, but it was associated with more severe patterns of the disease.

On the other hand, the majority of patients with positive serology who are not treated have the typical histological changes of CD[23,24]; however, those without villous atrophy (Group-II) account for one-third of CD cases[25,26]. In these cases, the false negative histology can be caused by the patchy distribution, which most often affects the distal or latent forms of the disease[4,27,28]. In our study, the benefit of CE in this context was very high given that CE allows observation of injuries consistent with villous atrophy in areas that are not accessible to the biopsy[29]. For some authors, the confirmation of CD in these groups of seropositive patients with a normal DB is the clinical and serological response to the GFD[20]. Patients who do not want to undergo endoscopy or for whom endoscopy is contraindicated (Group-III) constituted a minority. In these patients, CE is an alternative accepted method of diagnosis[30] and, in our experience indicates, is beneficial to half of the patients.

Finally, the majority of patients in which CE was requested in our series (Group-IV) were seronegative, lacked duodenal atrophy and had a positive HLA and a clinical presentation compatible with CD. The sensitivity of CE is lower in the absence of atrophy when endoscopic signs may remain unnoticed. However, the diagnostic impact in these cases was approximately 40%, and the main observation was intestinal atrophy. All new cases of NCGS and most other enteropathies belonged to this group. The distribution pattern observed using CE is frequently extensive enteropathy (duodenal continuing into patchy jejunal)[11]. This pattern occurred in 66.6% of the patients who exhibited symptoms in the proximal ID, and 11.1% had panenteric symptoms[31]. Similarly, in our study, the most frequent location of the atrophy was duodenal, followed by a widespread distribution, jejunal distribution, patchy distribution and occasionally an isolated ileal distribution. We found that almost 20% of the patients had atrophy unreachable by conventional endoscopy. The relevance of the spread of the enteropathy characteristics found using CE correlates to the ATG results but not to the clinical symptoms[10]. However, as in other studies[11], the percentage of the response to the GFD was higher in our patients who had villous atrophy.

As for the finding of ulcers in the UJ, they are distinguished from those found in other enteropathies or in patients without pathology because they are more numerous (≥ 5) and larger and distal[32]. Likewise, the distinction with other ulcerative diseases should be made in a context of adequate suspicion and after response to specific treatment.

Regarding the TI, the CE findings influenced the therapeutic approach in more than 70% of the patients, with the majority responding to the GFD. Similarly, a previous study reported that CE findings were consistent with histology findings in 78% of cases[33]. In our experience, there was a total agreement between the CE findings and the histology findings when the CE indicated normal results and 70% agreement for the diagnosis of atrophy.

### Weaknesses of the study

Our study has several limitations in addition to its retrospective design, which include the following: the use of different systems for CE, subjective CE criteria for CD diagnosis, with successive new criteria in histological classifications and clinical practice guidelines, additional endoscopic instrumentation (BAE), the lack of HLA assays for all cases, the broad time interval of the data collection, and the use of different investigators. Nevertheless, this study represents one of the largest series published to date for this type of patient, who are frequently encountered in normal clinical practice.

In conclusion, CE has a fundamental role with a high diagnosis impact in cases of misleading diagnosis for CD, with the CE modifying the clinical course, especially in cases with positive serology at baseline. In addition, the atrophy observed by CE has a high concordance with the results of subsequent histology and relates to the response to the GFD. This procedure is safe and useful, even when it indicates a normal result. The diagnostic performance along with the response to the GFD allowed a differentiation between CD, NCGS and other enteropathies. Therefore, our data suggest that in cases of misleading CD, CE can complement serology and biopsy in the early and differential diagnosis of this disease.

## ACKNOWLEDGMENTS

The study was recognized by the Enteroscopy and Capsule Endoscopy Spanish Society Group of the Spanish Society of Digestive Endoscopy.

## COMMENTS

### Background

Celiac disease (CD) is an autoimmune disease characterized by an increased immune response to gluten. Prevalence rates in populations in the America and Europe are estimated at 0.2%-1.0%. The diagnostic test is the histology of the small intestine through superior endoscopy demonstrating the presence of atrophy of the villi. The diagnosis of CD becomes a real challenge when all the factors of suspicion are not fulfilled. For this reason there is a growing interest in the role of the capsule endoscopy (CE) in this disease. Due to its ability to increase the intestinal image, it can detect villous atrophy compatible with celiac disease and other enteropathies or complications associated to this disease.

### Research frontiers

Studies have shown a utility of the endoscopic capsule for the diagnosis of celiac disease atrophy with sensitivity, specificity and positive and negative predictive values ​​of CE of 70%-100%, 64%-100%, 96%-100% and 71%-93%, respectively. There is currently no clinical practice guide that accurately defines the role of the CE in this context as the published series show a small number of cases. For this reason, the authors conducted this European multicenter study that allowed the inclusion of a greater number of cases, in order to define the appropriate use of CE in the suspicion of CD.

### Innovations and breakthroughs

CE has an important role with a high diagnostic impact in cases of misleading diagnosis for CD, with the CE modifying the clinical course, especially in cases with positive serology at baseline. In addition, the atrophy observed by CE has a high concordance with the results of subsequent histology and relates to the response to the gluten-free diet (GFD). This procedure is safe and useful, even when it indicates a normal result. The diagnostic performance along with the response to the GFD allowed a differentiation between CD, non-celiac gluten sensitivity and other enteropathies.

### Applications

Therefore, these data suggest that in cases of misleading CD, CE can complement serology and biopsy in the early and differential diagnosis of this disease.

### Terminology

CE is a non-invasive tool that displays the entire SB and is an alternative to duodenal biopsy in doubtful cases of celiac disease.

### Peer-review

This study shows the impact of CE on diagnosis and therapies for patients suspected of celiac disease.
